# OM-85 BV for primary prevention of recurrent airway infections: a pilot randomized, double-blind, placebo-controlled study

**DOI:** 10.31744/einstein_journal/2020AO5262

**Published:** 2020-02-19

**Authors:** Fátima Cleonice de Souza, Magáli Mocellin, Renata Ongaratto, Lidiane Alves de Azeredo Leitão, Frederico Orlando Friedrich, Victória d’A Silveira, Marcelo Comerlato Scotta, Paulo Márcio Pitrez, Leonardo Araújo Pinto

**Affiliations:** 1 Universidade de Santa Cruz do Sul Santa Cruz do SulRS Brazil Universidade de Santa Cruz do Sul , Santa Cruz do Sul , RS , Brazil .; 2 Pontifícia Universidade Católica do Rio Grande do Sul Porto AlegreRS Brazil Pontifícia Universidade Católica do Rio Grande do Sul , Porto Alegre , RS , Brazil .

**Keywords:** Respiratory tract infections/drug therapy, Placebo, Adjuvants, immunologic/therapeutic use, Child, Primary prevention

## Abstract

**Objective:**

To compare the frequency of respiratory tract infections in children treated with OM-85 BV and placebo during the 3-month therapy period, and observation for a further 3 months after treatment.

**Methods:**

A randomized, double-blind, placebo-controlled trial was conducted with 54 children (6 months to 5 years old) with no past history of recurrent respiratory infections attending daycare center. Family members were instructed to administer one capsule per day for 10 consecutive days, for 3 months of OM-85 BV or placebo. Telephone interviews were conducted every 30 days.

**Results:**

There was no significant difference in the number of respiratory infections between the groups. The mean number of respiratory tract infection in the OM-85 BV Group in the first 3 months was 0.92±0.87, and in the Placebo Group was 0.74±1.02, and at 6 months it was 1.62±1.47 and 1.03±1.34, respectively.

**Conclusion:**

OM-85 BV was not effective in the primary prevention of respiratory tract infections. Although most authors recommend the use of this immunostimulant in children with a history of recurrent respiratory infections, more studies are needed to define its usefulness in the primary prevention of respiratory infections in healthy children exposed to few risk factors.

## INTRODUCTION

Respiratory tract infections (RTIs) are a major cause of childhood morbidity and mortality. In developed countries, RTI are among the most frequent diseases, accounting for 20% of medical consultations and 75% of antibiotic prescriptions. ^1 , 2^ The high incidence of RTI in infants and preschool children is explained by increased exposure to respiratory pathogens by siblings and caregivers, by environmental factors and immaturity of the immune system. ^3^ Overcrowding conditions, such as that found in some daycare centers, absence or short period of breastfeeding, passive smoking and exposure to pollutants are among the risk factors for RTI in childhood. ^4 - 6^

It is widely accepted that the recurrence of RTI increases the predisposition to future respiratory problems. ^7 , 8^ Repeated lower RTI in the first 3 years of life have a positive association with wheezing until the age of 7 years. ^9^ In addition to the preventive measures already proven to reduce recurrent respiratory infections (RRI), as promoting breastfeeding, adequate vaccination of children, vaccination of pregnant women, hygiene measures and tobacco-free environment, bacterial immunomodulators have been proposed as preventive intervention. ^10 , 11^

OM-85 BV is an immunostimulant used to prevent RRI. This immunostimulant contains 3.5mg per capsule of a lyophilized fraction of alkaline lysis of 21 strains of 8 species of common respiratory tract pathogens: *Haemophilus influenza, Streptococcus pneumoniae, Klebsiella pneumoniae, Klebsiella ozaenae, Staphylococcus aureus, Streptococcus pyogenes, Streptococcus viridans* and *Neisseria catarrhalis* . ^1 , 12^

The mechanism of action of OM-85 BV was reviewed by Rozy et al. ^10^ This medication induces terminal maturation of human dendritic cells with increased *in vitro* capacity of stimulatory T cells. OM-85 BV produces an effect on innate immunity influencing macrophage and neutrophil activity, and production of pro-inflammatory cytokines (mainly interferon gamma − IFN-γ −, interleukin − IL-2, IL-1, IL-6, IL-8 and tumor necrosis factor alpha − TNF-α), as well as on the immune response regulated by lymphocytes and synthesis of immunoglobulins. ^10 , 13^

From the clinical point of view, studies have demonstrated safety and efficacy in use of bacterial immunostimulants. ^14 , 15^ In a systematic review, it was observed that children treated with OM-85 BV had significantly fewer cases of RRI. These data also suggest that the effect is greater in patients with increased risk of RRI or higher number of RRI prior to inclusion in the study. ^16^ The role of OM-85 BV in the primary prevention of respiratory infection still needs to be clarified.

## OBJECTIVE

To evaluate the effect of OM-85 BV on the incidence of airway infections in children with no history of recurrent respiratory infections, who attended daycare center, during treatment and during the observation period.

## METHODS

### Design and patients

This was a randomized, double-blind, placebo-controlled clinical trial. Children attending Kindergarten Margarida Aurora, at Santa Cruz do Sul (RS, Brazil) were included. Inclusion criteria were infants and children without previous history of RRI, aged between 6 months and 5 years. Exclusion criteria were children with cystic fibrosis, significant systemic disease (hepatitis and/or renal disease, malignancy), disorders of the immune system, suspected malabsorption, known allergy to the bacterial extract OM-85 BV, children submitted to major surgical procedures, such as traumatic, neurological, vascular and abdominal surgeries, up to 3 months before the start of the study, immunosuppressive therapy with corticosteroids for more than 7 days, or that were already in use of OM-85 BV or other immunostimulants.

### Randomization, intervention and data collection

Simple randomization was performed. The children were randomized in a ratio of 1:1 for one of the two groups, OM-85 BV or Placebo. These medications were distributed in three vials, each containing ten capsules. Family members were instructed verbally and in writing to administer one capsule (OM-85 BV or Placebo) per day, for 10 consecutive days, for 3 months, and the capsules should be diluted in 5mL of cold liquid. Both the OM-85 BV and placebo were prepared by a compounding pharmacy. Medication and placebo were packaged and identified by a code (A or B) known only to the pharmacist responsible for handling. Thus, it was not possible to distinguish them at any time during the study period, neither by patients nor by researchers.

The duration of the study was 7 months, and every 30 days telephone interviews were conducted with those responsible between April and September. Demographic data, prevalence of breastfeeding, history of passive smoking, signs of infection, number of visits to the emergency department, hospitalizations, use of antibiotics, episodes of wheezing, and adverse effects related to the intervention were collected in the telephone interviews. Recurrent respiratory infections were defined as the occurrence of three or more episodes of RTI within 6 months.

### Statistical and ethical issues

The sample size was calculated to provide a 50% decrease in mean RRI in the OM-85 BV Group compared to the Placebo Group, and to be statistically significant. Chi-squared test (categorical variables) and Sudents’s *t* -test (continuous variables) were used to compare the two groups. For variables with asymmetric distribution, non-parametric Mann-Whitney tests were used. Data were processed and analyzed using (SPSS). P-values of ≤0.05 were considered significant.

The protocol was approved by the Research Ethics Committee of *Pontifícia Universidade Católica do Rio Grande do Sul* (CAAE: 12548613.3.0000.5336; under opinion number 463.818) and authorized by the Municipal Education Department of the city of Santa Cruz do Sul. Parents of all children included in the study signed an Informed Consent Form.

## RESULTS

The study involved 54 children. Twenty-seven patients were from the OM-85 BV Group (cases) and 27 were from the Placebo Group. All patients completed the study. There were no significant differences between groups.

The characteristics of the subjects studied are shown in table 1 . Fifty-seven percent of the sample (n=31) were male, 12 (44.4%) in the OM-85 BV Group and 19 (70.4%) in the Placebo Group. In the OM-85 BV Group, the mean age was 3.6±1.3 years, and in the Placebo Group, it was 3.0±1.5 years. The age of admission to the daycare center was similar in both groups. Regarding breastfeeding and family history of smoking, there was no significant difference between patients in the OM-85 BV Group and in the Placebo Group.

**Table 1:** Characteristics of the sample

Characteristics	OM-85 BV Group (n=27)	Placebo Group (n=27)	p value
Male*	12 (44.4)	19 (70.4)	0.09
Age, years ^†^	3.6±1.3	3.04±1.5	0.15
Age at daycare, months ^†^	16.7±13.6	13.8±11.9	0.41
Breastfeeding*			0.76
Less than 1 month	5 (18.5)	7 (25.9)	
Exclusive up to 3 months	16 (59.3)	13 (48.1)	
Exclusive up to 6 months	6 (22.2)	7 (25.9)	
Family history of smoking*	11 (40.7)	13 (48.1)	0.78

Results expressed as n (%) or mean±standard deviation.

* χ ^2^ tests; ^†^ *t* test.

Regarding the number of respiratory infections (primary outcome), the mean in the OM-85 BV Group in the first 3 months was 0.92±0.87, and in the Placebo Group it was 0.74±1.02, and, at 6 months, it was 1.62±1.47 and 1.03±1.34, respectively. Antibiotic was used in 10 (37%) children of the OM-85 BV Group in the 3 months of treatment, and in 13 (48.1%) of the Placebo Group, and in the 6 months of follow-up, the use was 48.1% in both groups. The mean number of hospitalizations was very low in the study period, with no statistical significance, but only one (3.7%) child in the OM-85 BV Group was hospitalized in the 6-month period and three (11.1%) in the Placebo Group. The incidence of wheezing was the same in both groups, occurring in five (18.5%) children in the 3 months of treatment, and seven (25.9%) in the 6 months of follow-up ( Table 2 ). There were no reports of adverse events possibly associated with intervention in any of the groups studied.

**Table 2:** Number of respiratory infections, emergency visits, frequency of antibiotics usage, hospitalization and wheezing episodes

Outcomes	OM-85 BV (n=27)	Placebo (n=27)	p value
Number of respiratory infections, months*			
3	0.92±0.87	0.74±1.02	0.26
6	1.62±1.47	1.03±1.34	0.94
Number of emergency room consultations, months*			
3	0.81±1.21	1.29±1.48	0.17
6	1.77±1.80	2.03±1.76	0.58
Use of antibiotics, months ^†^			
3	10 (37.0)	13 (48.1)	0.58
6	13 (48.1)	13 (48.1)	1.00
Hospital admissions, months ^†^			
3	0	3 (11.1)	0.23
6	1 (3.7)	3 (11.1)	0.61
Wheezing, months ^†^			
3	5 (18.5)	5 (18.5)	1.00
6	7 (25.9)	7 (25.9)	1.00

Results expressed as n (%) or mean±standard deviation.

* *t* test; ^†^ χ ^2^ tests.

## DISCUSSION

Recurrent respiratory infections are common in pediatric healthy young patients, and may be triggered by different types of virus and bacteria. ^11 , 14^ In this study, with children without history of RRI, no beneficial effect was observed with the use of OM-85 BV. There was no significant difference in the number of respiratory infections nor in the use of antibiotics, visits to the emergency department, hospital admissions, and episodes of wheezing between the groups.

Most studies in children with RRI showed significant effect of OM-85 BV in reducing the number of episodes of infection (primary outcome), having a potential pharmacoeconomic impact, particularly in nurseries and schools. ^17 , 18^ Schaad et al., in a multicenter trial with OM-85 BV in 232 children, found that, in the treated group, children had significantly less RTI as compared to patients receiving placebo. ^19^ A systematic review analyzing 8 randomized studies with 851 children also found a significant reduction in RTI (32% of patients in the group treated with OM-85 BV had RTI, as compared to 58% in the Placebo Group), being the greater effect in patients with increased risk of RRI. ^16^ Jara-Pérez et al., observed better results in the group receiving OM-85 BV than in the Placebo Group, in relation to the number of RTI and disease duration, as well as absences at school and use of antibiotics. ^20^

Esposito et al., evaluated the efficacy of OM-85 BV when administered for 2 consecutive years. In the group of children treated with OM-85 BV, the number of patients who did not experience any new episode of RTI, as well as the number of RTI, wheezing episodes, medical visits, and prescribed antibiotic courses, were significantly lower than that in the group not treated with OM-85 BV. The study suggest that OM-85 BV can effectively and safely reduce the risk of new infectious episodes in children with recurrent RTI, and that a second-year course of lysate administration can be useful to maintain protection. ^15^

Our study confirms safety in OM-85 BV administration, due to the absence of relevant side effects. The cost-benefit ratio is clearly in favor of use of OM-85 BV in secondary prevention, and its use may be recommended only in children with a history of frequent RRI during the endemic period of respiratory infections. ^12^ Studies show that the higher the number of previous RTI, the greater the effect size. ^16^ Despite this, there are still questions to be answered about the long-term efficacy of immunostimulants, efficacy according to the type RTI (upper or lower) and the role of immunostimulants in primary prevention.

Previous studies evaluating the efficacy of OM-85 BV included only children with history of RRI. ^7 , 16 , 17^ In our study, we did not use this data as inclusion criterion. Children were included regardless of the past history of infections. In addition, the children attended a daycare center with good infrastructure and good hygiene conditions, which had an adequate number of caregivers and rooms separated by age group. Contact precautions, such as handwashing and reduced number of children per room, as well as keeping children with any health problems away from the classroom, were implemented in the daycare. This reinforces the finding that the group studied is not necessarily the group of patients who could benefit most from the use of OM-85 BV.

The low frequency of respiratory infections seems to have been the determining factor for the negative results. However, this is in line with the main finding of the study, *i.e* ., the lack of benefits of using OM-85 BV in samples at low risk for respiratory infections. Therefore, the outcome of our study is important to avoid unnecessary use of such medication in children without prior history of RRI.

The study has some limitations. It is a pilot study and the sample size is one of the limitations of the study. However, there was no trend indicating any difference in favor of the intervention, which makes a false negative result very unlikely. Also, uncertain adherence to prescribed treatment is a possible limitation in intervention studies. However, in our study, this factor was widely considered. In telephone interviews, which occurred every 30 days during the 7-month study period, information regarding adherence to treatment was questioned and computed.

## CONCLUSION

OM-85 BV was not effective in the primary prevention of recurrent respiratory infections and did not show statistically significant difference in antibiotic use, visits to the emergency department, hospitalizations, and wheezing episodes. Therefore, more studies are needed to define its usefulness in preventing respiratory tract infections in previously healthy children exposed to risk factors.
