# Supplementary material for: OM-85 BV for primary prevention of recurrent airway infections: a pilot randomized, double-blind, placebo-controlled study
Source: Einstein (Sao Paulo). 2020 Feb 19;18:eAO5262. doi: 10.31744/einstein_journal/2020AO5262 (PMC7032885; doi:10.31744/einstein_journal/2020AO5262)
Supplement: Translation

# Translation: OM-85 BV para prevenção primária de infecções recorrentes das vias aéreas: estudo piloto, randomizado, duplo-cego, placebo-controlado

**Authors:** Fátima Cleonice de Souza, Magáli Mocellin, Renata Ongaratto, Lidiane Alves de Azeredo Leitão, Frederico Orlando Friedrich, Victória d’A Silveira, Marcelo Comerlato Scotta, Paulo Márcio Pitrez, Leonardo Araújo Pinto

**Affiliations:** 1 Brasil Universidade de Santa Cruz do Sul, Santa Cruz do Sul, RS, Brasil .; 2 Brasil Pontifícia Universidade Católica do Rio Grande do Sul, Porto Alegre, RS, Brasil .

**Keywords:** Infecções respiratórias/tratamento farmacológico, Placebos, Adjuvantes imunológicos/ uso terapêutico, Criança, Prevenção primária

## Abstract

**Objetivo:**

Comparar a frequência de infecções do trato respiratório em crianças tratadas com OM-85 BV e placebo durante o período de terapia de 3 meses, e observação por mais 3 meses após o tratamento.

**Métodos:**

Foi realizado estudo randomizado, duplo-cego, controlado por placebo com 54 crianças (6 meses a 5 anos) sem história prévia de infecções respiratórias recorrentes, que frequentavam creches. Os membros da família foram instruídos a administrar uma cápsula por dia durante 10 dias consecutivos, durante 3 meses, de OM-85 BV ou placebo. Entrevistas telefônicas foram realizadas a cada 30 dias.

**Resultados:**

Não houve diferença significativa no número de infecções respiratórias entre os grupos. O número médio de infecções do trato respiratório no Grupo OM-85 BV nos primeiros 3 meses foi de 0,92±0,87 e, no Grupo Placebo, de 0,74±1,02, e aos 6 meses foi de 1,62±1,47 e 1,03±1,34, respectivamente.

**Conclusão:**

O OM-85 BV não foi eficaz na prevenção primária de infecções do trato respiratório. Embora a maioria dos autores recomende o uso deste imunoestimulante em crianças com história de infecções respiratórias recorrentes, mais estudos são necessários para definir sua utilidade na prevenção primária de infecções respiratórias em crianças saudáveis expostas a poucos fatores de risco.

## INTRODUÇÃO

As infecções do trato respiratório (ITR) são uma das principais causas de morbidade e mortalidade na infância. Em países desenvolvidos, elas estão entre as doenças mais frequentes, representando 20% das consultas médicas e 75% das prescrições de antibióticos.^1 , 2^A alta incidência de ITR em lactentes e crianças pré-escolares é explicada pela maior exposição a patógenos respiratórios de irmãos e cuidadores, assim como fatores ambientais e imaturidade do sistema imunológico.^3^Condições de superlotação, como em algumas creches, ausência ou curta duração de aleitamento materno, fumo passivo e exposição a poluentes estão entre os fatores de risco para ITR na infância.^4 - 6^

Sabe-se que a recorrência de ITR aumenta a predisposição a problemas respiratórios futuros.^7 , 8^Infecções repetitivas do trato respiratório inferior nos primeiros 3 anos de vida estão positivamente associadas à ocorrência de sibilos até os 7 anos de idade.^9^Além das medidas preventivas, que comprovadamente reduzem infecções respiratórias recorrentes (IRR), como incentivo ao aleitamento materno, vacinação adequada das crianças, vacinação de gestantes, medidas de higiene e ambientes livres de tabaco, propõe-se o uso de imunomoduladores bacterianos como intervenção preventiva.^10 , 11^

O OM-85 BV é um medicamento imunoestimulante usado na prevenção de IRR. Este imunoestimulante contém 3,5mg por cápsula de uma fração liofilizada da lise alcalina de 21 cepas de 8 espécies de patógenos comuns do trato respiratório: *Haemophilus influenza, Streptococcus pneumoniae, Klebsiella pneumoniae, Klebsiella ozaenae, Staphylococcus aureus, Streptococcus pyogenes, Streptococcus viridans* e *Neisseria catarrhalis* .^1 , 12^

O mecanismo de ação do OM-85 BV foi analisado por Rozy et al.^10^O fármaco induz a maturação terminal de células dendríticas humanas, com maior capacidade *in vitro* de linfócitos T estimuladores. O OM-85 BV tem efeito sobre a imunidade inata, influenciando na atividade de macrófagos e neutrófilos, e na produção de citocinas pró-inflamatórias (principalmente interferon-gama − IFN-γ −, interleucina – IL − 2, IL-1, IL-6, IL-8 e fator de necrose tumoral alfa − TNF-α), bem como na resposta imune regulada por linfócitos e na síntese de imunoglobulinas.^10 , 13^

Do ponto de vista clínico, estudos já demonstraram a segurança e a eficácia do uso de imunoestimulantes bacterianos.^14 , 15^Revisão sistemática mostrou que crianças tratadas com OM-85 BV tiveram número significativamente menor de IRR. Esses dados também sugerem que o efeito é maior em pacientes com risco aumentado de IRR, ou com maior número de IRR antes da inclusão no estudo.^16^O papel do OM-85 BV na prevenção primária de infecções respiratórias ainda precisa ser esclarecido.

## OBJETIVO

Avaliar o efeito do OM-85 BV na incidência de infecções das vias aéreas em crianças sem história de infecções respiratórias recorrentes, frequentadoras de creches, durante os períodos de tratamento e de observação.

## MÉTODOS

### Desenho e pacientes

Trata-se de estudo clínico randomizado, duplo-cego, controlado por placebo. O estudo incluiu crianças da creche Margarida Aurora, em Santa Cruz do Sul (RS). Os critérios de inclusão foram lactentes e crianças sem história prévia de IRR, com idade entre 6 meses e 5 anos. Os critérios de exclusão foram crianças com fibrose cística, doença sistêmica significativa (hepatite e/ou doença renal, neoplasia maligna), transtornos do sistema imunológico, suspeita de má absorção, alergia conhecida ao extrato bacteriano OM-85 BV, crianças submetidas a cirurgias de grande porte, como de trauma, neurológicas, vasculares e abdominais, nos 3 meses anteriores ao início do estudo, terapia imunossupressora com corticoides por mais de 7 dias, ou que já estivessem sendo tratadas com OM-85 BV ou outros imunoestimulantes.

### Randomização, intervenção e coleta de dados

O estudo usou randomização simples. As crianças foram randomizadas em proporção de 1:1 para um dos dois grupos, OM-85 BV ou Placebo. Os medicamentos foram distribuídos em três frascos, cada um com dez cápsulas. Os familiares foram instruídos verbalmente e por escrito a administrarem uma cápsula (OM-85 BV ou placebo) por dia, por 10 dias consecutivos, por 3 meses, sendo que as cápsulas deveriam ser diluídas em 5mL de líquido frio. Tanto o OM-85 BV quanto o placebo foram preparados em farmácia de manipulação. O fármaco e o placebo foram embalados e identificados por um código (A ou B), conhecido somente pelo farmacêutico responsável pela manipulação, de modo que nem os pacientes, nem os pesquisadores conseguiram distingui-los, em nenhum momento durante o estudo.

A duração do estudo foi de 7 meses, e a cada 30 dias eram conduzidas entrevistas telefônicas com os responsáveis, entre abril e setembro. Dados demográficos, prevalência de aleitamento materno, história de fumo passivo, sinais de infecção, número de visitas ao pronto atendimento, internações, uso de antibióticos, episódios de sibilância e efeitos adversos relacionados à intervenção foram coletados nas entrevistas telefônicas. Infecções respiratórias recorrentes foram definidas como ocorrência de três ou mais episódios de ITR em um período de 6 meses.

### Estatística e aspectos éticos

O tamanho da amostra foi calculado para proporcionar diminuição de 50% no número médio de IRR no Grupo OM-85 BV, em comparação ao Grupo Placebo, alcançando significância estatística. O teste do χ^2^(variáveis categóricas) e o teste *t* de Student (variáveis contínuas) foram usados para comparar os dois grupos. Para variáveis com distribuição assimétrica, foram usados testes não paramétricos de Mann-Whitney. Os dados foram processados e analisados pelo programa (SPSS). Valores de p≤0,05 foram considerados significativos.

O protocolo foi aprovado pelo Comitê de Ética em Pesquisa da Pontifícia Universidade Católica do Rio Grande do Sul (CAAE: 12548613.3.0000.5336; sob o parecer 463.818) e autorizado pela Secretaria Municipal de Educação da cidade de Santa Cruz do Sul. Os pais de todas as crianças incluídas no estudo assinaram um Termo de Consentimento Livre e Esclarecido.

## RESULTADOS

O estudo incluiu 54 crianças. O Grupo OM-85 BV incluiu 27 pacientes, e os outros 27 ficaram no Grupo Placebo. Todos os pacientes concluíram o estudo. Não houve diferenças significativas entre os grupos.

As características dos participantes são apresentadas na tabela 1 . Dentre os participantes, 57% (n=31) eram do sexo masculino, 12 (44,4%) no Grupo OM-85 BV e 19 (70,4%) no Placebo. No Grupo OM-85 BV, a idade média foi de 3,6±1,3 anos, e no Placebo, 3,0±1,5 anos. A idade de ingresso na creche foi semelhante nos dois grupos. Em relação ao aleitamento materno e à história familiar de tabagismo, não houve diferença significativa entre pacientes dos Grupos OM-85 BV e Placebo.

**Tabela 1:** Características da amostra

Características	Grupo OM-85 BV (n=27)	Grupo Placebo (n=27)	Valor de p
Sexo masculino*	12 (44,4)	19 (70,4)	0,09
Idade, anos^†^	3,6±1,3	3,04±1,5	0,15
Idade na creche, meses^†^	16,7±13,6	13,8±11,9	0,41
Aleitamento materno*			0,76
Menos de 1 mês	5 (18,5)	7 (25,9)	
Exclusivo até 3 meses	16 (59,3)	13 (48,1)	
Exclusivo até 6 meses	6 (22,2)	7 (25,9)	
História familiar de tabagismo^†^	11 (40,7)	13 (48,1)	0,78

Resultados expressos como n (%) ou média±desvio padrão.

* Teste do χ^2^;^†^teste *t* .

Em relação ao número médio de infecções respiratórias (desfecho primário), a média no Grupo OM-85 BV nos primeiros 3 meses foi de 0,92±0,87, e no Grupo Placebo foi de 0,74±1,02, e, aos 6 meses, foi de 1,62±1,47 e 1,03±1,34, respectivamente. Antibióticos foram usados por 10 (37%) crianças do Grupo OM-85 BV nos 3 meses de tratamento e 13 (48,1%) do Grupo Placebo, e nos 6 meses de acompanhamento, o uso foi de 48,1% nos dois grupos. O número médio de internações foi muito baixo durante o período de estudo, sem significância estatística, mas apenas uma (3,7%) criança no Grupo OM-85 BV foi internada no período de 6 meses, e 3 (11,1%) do Grupo Placebo. A incidência de sibilância foi a mesma nos dois grupos, tendo ocorrido em 5 (18,5%) crianças nos 3 meses de tratamento e em 7 (25,9%) nos 6 meses de acompanhamento ( Tabela 2 ). Não houve relatos de eventos adversos possivelmente associados à intervenção em nenhum dos grupos estudados.

**Tabela 2:** Número de infecções respiratórias, visitas ao pronto atendimento, frequência de uso de antibióticos, internação e episódios de sibilos

Desfechos	Grupo OM-85 BV (n=27)	Grupo Placebo (n=27)	Valor de p
Número de infecções respiratórias, meses*			
3	0,92±0,87	0,74±1,02	0,26
6	1,62±1,47	1,03±1,34	0,94
Número de visitas ao pronto-atendimento, meses*			
3	0,81±1,21	1,29±1,48	0,17
6	1,77±1,80	2,03±1,76	0,58
Uso de antibióticos, meses^†^			
3	10 (37,0)	13 (48,1)	0,58
6	13 (48,1)	13 (48,1)	1,00
Internações hospitalares, meses^†^			
3	0	3 (11,1)	0,23
6	1 (3,7)	3 (11,1)	0,61
Sibilância, meses^†^			
3	5 (18,5)	5 (18,5)	1,00
6	7 (25,9)	7 (25,9)	1,00

Resultados expressos como n (%) ou média±desvio padrão.

* Teste *t* ;^†^teste do χ^2^.

## DISCUSSÃO

As IRR são comuns em pacientes pediátricos saudáveis e podem ser desencadeadas por diversos tipos de vírus e bactérias.^11 , 14^Neste estudo, em crianças sem história de IRR, nenhum benefício foi observado com o uso de OM-85 BV. Não houve diferença significativa no número de infecções respiratórias nem no uso de antibióticos, visitas ao pronto atendimento, internações hospitalares e episódios de sibilância entre os grupos.

A maioria dos estudos clínicos em crianças com IRR mostra efeito considerável do OM-85 BV na redução do número de episódios infecciosos (desfecho primário), com possível impacto farmacoeconômico, principalmente em creches e escolas.^17 , 18^Schaad et al., em estudo multicêntrico com OM-85 BV em 232 crianças, constataram que, no grupo de tratamento, as crianças tiveram número significativamente menor de ITR, comparadas às que receberam placebo.^19^Revisão sistemática de oito estudos randomizados com 851 crianças também encontrou redução significativa nas ITR (32% dos pacientes no Grupo OM-85 BV tiveram ITR, em comparação a 58% no Grupo Placebo), sendo que o maior efeito foi observado em crianças com maior risco para IRR.^16^Jara-Pérez et al., observaram resultados superiores no grupo tratado com OM-85 BV em comparação ao placebo, em relação ao número e à duração das ITR, bem como faltas na escola e uso de antibióticos.^20^

Esposito et al., avaliaram a eficácia do OM-85 BV quando administrado por 2 anos consecutivos. No grupo de crianças tratadas com OM-85 BV, o número de pacientes que não apresentou episódio de ITR, bem como o número de ITR, episódios de sibilância, consultas médicas e cursos prescritos de antibióticos, foi significativamente menor do que no grupo não tratado com OM-85 BV. O estudo sugere que OM-85 BV consegue reduzir, de maneira efetiva e segura, o risco de novos episódios infecciosos em crianças com ITR recorrentes, e um curso de 2 anos de administração do lisado pode ser útil para manter a proteção.^15^

Nosso estudo confirma a segurança da administração de OM-85 BV, devido à ausência de efeitos colaterais relevantes. O custo-benefício claramente favorece o uso do OM-85 BV na prevenção secundária, e seu uso pode ser recomendado apenas em crianças com história de IRR frequentes durante o período endêmico de infecções respiratórias.^12^Estudos mostram que, quanto maior o número de ITR prévias, maior o efeito.^16^Mesmo assim, ainda existem perguntas não respondidas relativas à eficácia dos imunoestimulantes no longo prazo, à eficácia de acordo com o tipo de ITR (superior ou inferior) e ao papel dos imunoestimulantes na prevenção primária.

Estudos anteriores que investigaram a eficácia do OM-85 BV incluíram apenas crianças com história de IRR.^7 , 16 , 17^Neste estudo, esses dados não foram usados como critério de inclusão. As crianças foram incluídas independentemente de qualquer história prévia de infecções. Além disso, as crianças frequentavam creche com boa infraestrutura e boas condições de higiene, com número adequado de cuidadores e salas separadas por faixa etária. Precauções de contato, como lavagem das mãos e menor número de crianças por sala, assim como afastamento temporário de crianças com problemas de saúde, eram implementadas na creche. Isso reforça a conclusão de que o grupo estudado não é necessariamente o grupo de pacientes que mais poderia se beneficiar do uso do OM-85 BV.

A baixa frequência de infecções respiratórias parece ter sido fator determinante para os resultados negativos. Entretanto, isso está de acordo com a principal conclusão do estudo, ou seja, a ausência de benefício com o uso de OM-85 BV em amostras com baixo risco para infecções respiratórias. Portanto, o desfecho deste estudo é importante para evitar o uso desnecessário desses medicamentos em crianças sem história pregressa de IRR.

O estudo tem algumas limitações. Trata-se de estudo piloto, e o tamanho da amostra é uma de suas limitações. Entretanto, não houve tendência indicando qualquer diferença que favorecesse a intervenção, o que faz com que um resultado falso-negativo seja muito improvável. Além disso, a incerteza sobre a adesão ao tratamento prescrito é uma limitação possível em estudos de intervenção. No entanto, neste estudo, este fator foi amplamente considerado. Nas entrevistas telefônicas, que ocorreram a cada 30 dias durante o período de 7 meses do estudo, informações sobre a adesão ao tratamento foram coletadas e computadas.

## CONCLUSÃO

O OM-85 BV não foi eficaz na prevenção primária de infecções respiratórias recorrentes e não demonstrou diferença estatisticamente significativa no uso de antibióticos, visitas à emergência, internações e episódios de sibilância. Mais estudos são necessários para definir sua utilidade na prevenção de infecções do trato respiratório em crianças previamente saudáveis, expostas a fatores de risco.
